# Disseminated Kaposi sarcoma presenting as an endophytic renal mass with concurrent primary effusion lymphoma: A case report

**DOI:** 10.1016/j.idcr.2025.e02310

**Published:** 2025-07-03

**Authors:** Shamieh Banihani, Alexandra C. Greb, Abhinav R. Thummala, Jonathan R. Said, Inderpreet S. Saini

**Affiliations:** a Department of Internal Medicine, UCLA-Olive View Medical Center, Sylmar, CA, USA; b Department of Internal Medicine, University of California, Los Angeles (UCLA) David Geffen School of Medicine, Los Angeles, CA, USA; c Department of Pathology, University of California, Los Angeles (UCLA) David Geffen School of Medicine, Los Angeles, CA, USA

**Keywords:** Kaposi Sarcoma, Human herpesvirus-8 (HHV-8), Human immunodeficiency virus (HIV), Primary effusion lymphoma (PEL)

## Abstract

Primary effusion lymphoma (PEL) is a rare, aggressive B-cell lymphoma associated with human herpesvirus-8 (HHV-8) and immunodeficiency, notably in untreated human immunodeficiency virus (HIV). Kaposi sarcoma (KS) is also an HHV-8-driven malignancy. Most cases of KS present with cutaneous manifestations, with less frequent visceral involvement. We present the case of a 63-year-old male with untreated HIV/AIDS who developed KS presenting as an endophytic renal mass with lymphovascular invasion and concurrent HHV-8-positive PEL. Biopsies confirmed KS in the kidney and PEL in the pleural fluid. Given profound immunosuppression, he was started on combination antiretroviral therapy and chemotherapy. This case represents a rare presentation of KS and highlights the aggressive course of HHV-8-driven malignancies in the setting of advanced HIV. Delayed antiretroviral initiation likely contributed to disease progression, emphasizing the critical role of early HIV treatment.

## Highlights

• HHV-8 (aka Kaposi sarcoma associated herpesvirus, KSHV) is a cause of Kaposi sarcoma (KS).
• Kaposi sarcoma often presents as characteristic cutaneous vascular lesions.
• KS can more rarely localize in viscera.
• Our case is one of the only published cases of KS presenting as a renal mass.

## Introduction

Individuals with human immunodeficiency virus (HIV) have a higher risk of aggressive B-cell malignancies, including Burkitt lymphoma and diffuse large B-cell lymphoma [1]. Among these is primary effusion lymphoma (PEL), a rare type of non-Hodgkin lymphoma that is strongly associated with immunodeficiency, including those individuals with advanced HIV infection and associated profound immunosuppression [2]. PEL is particularly notable for presentation as massless effusions in serous cavities, notably the pleural cavity, and its aggressive nature marked by an extremely poor prognosis. PEL, along with Kaposi sarcoma, is driven by herpes virus-8 (HHV-8), an oncogenic virus that disrupts cell cycle regulation and apoptosis, among other antioncogenic mechanisms [3]. In this case, we present a patient with HIV and profound immunosuppression found to have Kaposi sarcoma with renal involvement and concurrent HHV-8 positive PEL.

## Case presentation

**Fig. 1:**
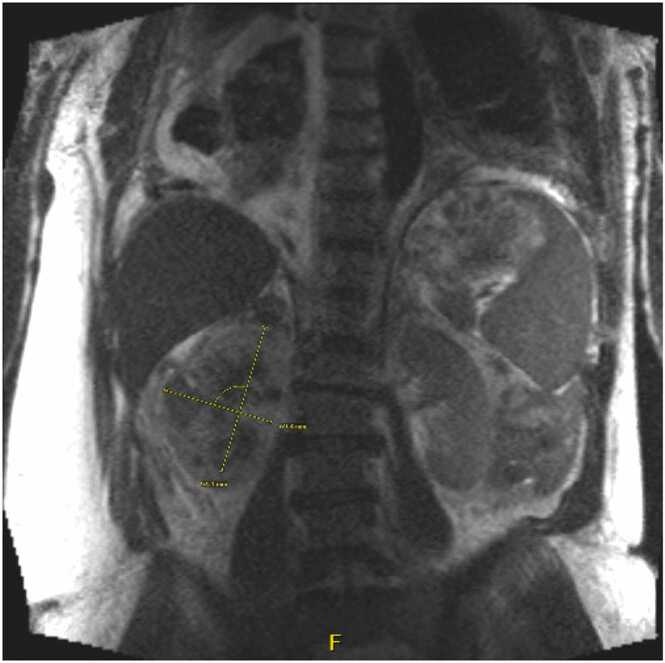
MRI abdomen coronal demonstrating a 9 cm soft tissue mass encasing the kidney with extension along the renal vasculature. Abbreviations: MRI, magnetic resonance imaging.

A 63-year-old male patient with a history of right popliteal entrapment syndrome and benign prostatic hyperplasia presented with shortness of breath and right lower extremity pain and swelling. Ten years prior to admission, the patient was reportedly diagnosed with HIV but remained untreated due to fear of medication side effects. On admission, his CD4 count was 148 cells/mm3, and his HIV-1 viral load was 66,000 copies/mL. Given his vascular history, he underwent computed tomography angiography of the chest, which revealed a new, large right pleural effusion, new right renal mass encasing critical vascular structures, and multistation abdominal lymphadenopathy (Fig. 1).

**Fig. 2:**
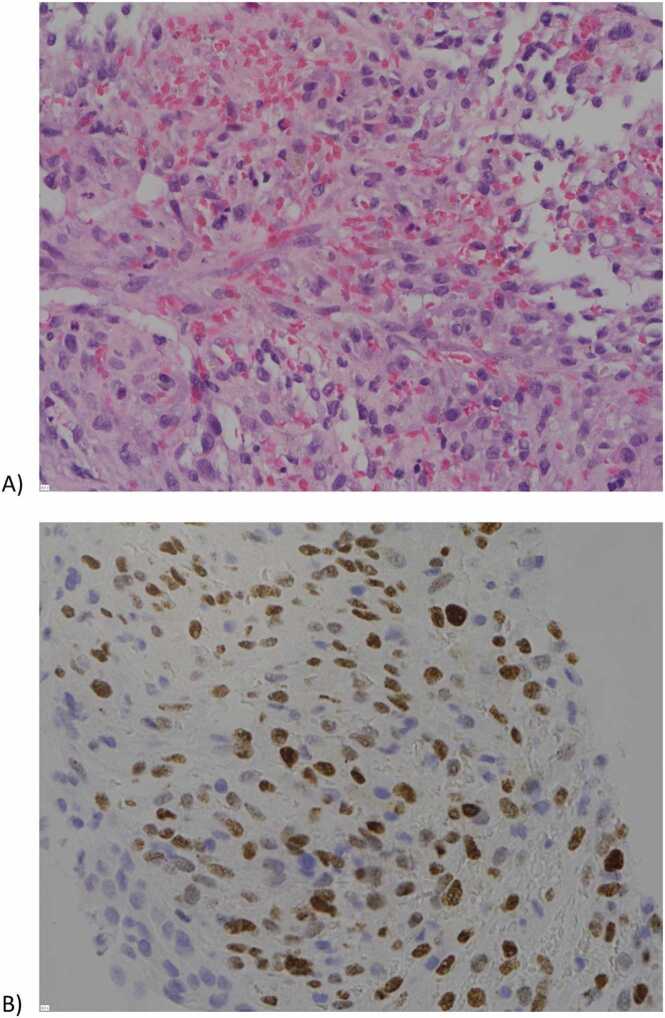
A) H&E-stained section of right renal tissue showing infiltration of the renal parenchyma by malignant spindle cells, and B) positive LANA immunostaining indicative of Kaposi sarcoma-associated herpesvirus, consistent with the diagnosis of Kaposi sarcoma. Abbreviations: H&E, Hematoxylin and Eosin; HHV-8, Human Herpesvirus 8; LANA, latency-associated nuclear staining.

Biopsies of the left axillary lymph node and the right renal mass were performed and confirmed Kaposi sarcoma (Fig. 2). Pulmonology was consulted for management of the large pleural effusion, and the patient underwent chest tube placement with pleural fluid cytology diagnostic for primary effusion lymphoma. Pleural fluid studies were also notably positive for HHV-8 and weakly positive for Epstein Barr virus. Infectious Disease was consulted, and patient was initiated on combination antiretroviral therapy with combination bictegravir, emtricitabine & tenofovir alafenamide. Hematology/Oncology recommended initiation of EPOCH chemotherapy for primary effusion lymphoma [4]. Additional issues addressed during hospitalization included anemia managed with transfusions, thrombocytopenia, and acute kidney injury (superimposed on history of chronic kidney disease) exacerbated by a compressive renal mass. The patient’s right lower extremity pain and swelling were attributed to lymphadenopathy and possible venous congestion, with no acute surgical intervention required. Prior to discharge, his chest tube was converted to a PleurX catheter. He was discharged with plans for close follow-up with Infectious Disease, Pulmonology, Hematology/Oncology, and Nephrology to monitor his complex medical conditions and ensure continued care. In summary, this patient was noted to have Kaposi sarcoma and primary effusion lymphoma in the setting of untreated HIV/AIDS.

## Discussion

Kaposi sarcoma is a vascular malignancy associated with HHV-8, an oncogenic virus and aetiologic agent of certain HIV-associated malignancies. HHV-8 encodes oncogenic proteins that contribute to malignancy onset by disrupting cell cycle regulation and apoptosis, and other anti-oncogenic mechanisms [5]. The HHV-8 virus is often a dormant pathogen without clinical manifestations in healthy individuals. However, among patients with immunosuppression, reactivation leads to proliferation and malignancy. In particular, HHV-8 infection of endothelial cells and reactivation with subsequent proliferation results in cutaneous vascular lesions characteristic of Kaposi Sarcoma [6]. Kaposi sarcoma rarely presents as a solid organ mass in any context. Well-documented cases of this malignancy predominantly involve cutaneous lesions, with only rare instances of disseminated disease or organ masses causing lymphovascular obstruction, as seen in this patient [7]. A review of the literature reveals that renal involvement by Kaposi sarcoma has been described almost exclusively in post-renal transplant patients receiving immunosuppressive therapy, and even in this setting, cases remain exceptionally rare [8]. To our knowledge, our case represents one of the only published instance of Kaposi sarcoma presenting as a renal mass in a non-renal transplant patient. Furthermore, descriptions of Kaposi sarcoma manifesting as a primary renal mass with non-specific lymphadenopathy are exceedingly scarce.

Further complicating this patient’s case is the diagnosis of PEL, which is also driven by HHV-8 [9]. In our patient with a very low CD4 count and high HIV viral load the co-occurrence of KS and PEL is associated with immunosuppression due poorly-controlled HIV infection. This patient’s advanced and disseminated disease was likely multifactorial, however, one important consideration is the initial delay in initiating antiretroviral treatment after diagnosis 10 years ago. This delay emphasizes the importance of appropriate counseling and education on antiretroviral therapy after an HIV diagnosis, particularly given the widespread tolerability and efficacy of these medications. Patients with PEL are conventionally treated with anthracycline-containing chemotherapy regimens, however further targeted therapies [e.g. lena/pomalidomide, PDl-1 inhibition] are also being studied, and survival expectations have continued to improve [10], [11]. Referral of patients with complex and multiple KAD to specialized HIV oncology care teams is crucial.

In this case, we describe a patient with history notable HIV that was left untreated for 10 years found to have disseminated Kaposi Sarcoma with renal mass and concomitant PEL causing malignant pleural effusion. While KAD do occur in individuals with well managed HIV infection this case exemplifies the excess risk borne associated with poorly controlled HIV infection: early initiation of antiretroviral therapy and optimal HIV control can in part manage the risk of KAD with many patients now living long, healthy lives despite a diagnosis of HIV.

## CRediT authorship contribution statement

**Inderpreet S. Saini:** Writing – original draft, Writing – review & editing, Supervision. **Jonathan R. Said:** Methodology. **Shamieh Banihani:** Writing – review & editing, Methodology, Formal analysis, Writing – original draft, Investigation, Conceptualization. **Abhinav R. Thummala:** Writing – original draft, Formal analysis, Writing – review & editing, Methodology. **Alexandra C. Greb:** Writing – original draft, Investigation, Writing – review & editing, Methodology, Formal analysis.

## Ethical approval

The study is a case report and only information from the patient's file was used. No intervention was performed, thus it does not have approval from the ethics committee.

## Consent

Written informed consent is obtained from the patient. A copy of the written consent is available for review by the Editor-in-Chief of this journal on request.

## Funding

This research did not receive any specific grant from funding agencies in the public, commercial, or not-for-profit sectors.

## Declaration of Competing Interest

The authors declare that they have no known competing financial interests or personal relationships that could have appeared to influence the work reported in this paper.

## References

[1] Gloghini A., Dolcetti R., Carbone A. (2013). Lymphomas occurring specifically in HIV-infected patients: from pathogenesis to pathology. Antigens Lymphoma Dev.

[2] Nador R.G., Cesarman E., Chadburn A. (1996). Primary effusion lymphoma: a distinct clinicopathologic entity associated with the Kaposi’s sarcoma-associated herpes virus. Blood.

[3] Schulz T.F., Cesarman E. (2015). Kaposi sarcoma-associated herpesvirus: mechanisms of oncogenesis. Eng Viral Resist Virus Cancer.

[4] Patel R., Lurain Kathryn, Yarchoan Robert, Ramaswami R. (2023). Clinical management of Kaposi sarcoma herpesvirus-associated diseases: an update on disease manifestations and treatment strategies. Expert Rev Anti Infect Ther.

[5] Etemad S.A., Dewan A.K. (2019). Kaposi sarcoma updates. Inter Dermatol Oncol.

[6] Mohseni Afshar Z., Goodarzi A., Emadi S.N. (2023). A comprehensive review on HIV-associated dermatologic manifestations: from epidemiology to clinical management. Int J Microbiol.

[7] Kawakami N., Namkoong H., Shimoda M., Kotani H., Fujiwara H., Hasegawa N. (2020). Hidden disseminated extracutaneous AIDS-related Kaposi sarcoma. IDCases.

[8] El-Agroudy A.E., El-Baz M.A., Ismail A.M., Ali-El-Dein B., Shehab El-Dein A.B., Ghoneim M.A. (2003). Clinical features and course of Kaposi’s sarcoma in Egyptian kidney transplant recipients. Am J Transpl.

[9] Volesky-Avellaneda K.D., Luo Q., Amaswami R.R., et al. Primary effusion lymphoma in people with and without HIV infection in the United States. *AIDS*. Published online 9900. 〈https://journals.lww.com/aidsonline/fulltext/9900/primary_effusion_lymphoma_in_people_with_and.647.aspx〉. 10.1097/QAD.0000000000004154 PMC12122224 39945621

[10] Berber I., Altaca G., Aydin C. (2005). Kaposi’s sarcoma in renal transplant patients: predisposing factors and prognosis. Transpl Proc.

[11] Lurain K., Ramaswami R., Oksenhendler E. (2025). Primary effusion lymphoma prognostic score (PEL-PS): a validated international prognostic score in HIV-associated primary effusion lymphoma. Am J Hematol.

